# Review of the *Parasa undulata* (Cai, 1983) species group with the first conifer-feeding larva for Limacodidae and descriptions of two new species from China and Taiwan (Lepidoptera, Limacodidae)

**DOI:** 10.3897/zookeys.345.6179

**Published:** 2013-10-29

**Authors:** Shipher Wu, Weichun Chang

**Affiliations:** 1 Department of Entomology, National Taiwan University, Taipei, Taiwan. Address: 27, Lane 113, Sec. 4, Roosevelt Rd., Taipei, Taiwan; 2 Forestry Bureau, Council of Agriculture Executive Yuan, No. 2, Hangchou S. Rd., Sec. 1, Taipei, Taiwan

**Keywords:** Conifer-feeder, Limacodidae, *Parasa*, new species, *Picea*, Pinaceae, Taiwan

## Abstract

Although the caterpillars are well-known for the stings and magnificent coloration, the systematics of Limacodidae is historically neglected and chaotic due to the difficulty in matching the larval with adult stages as well as the very conservative and convergent adult morphology. One of the biggest taxonomic problems surrounds a collective group from Southeastern Asia, termed the “green limacodid moths”, which harbours at least 90 species placed in the genus *Parasa* Walker, 1859 and 14 “subunits”. The *P. undulata* group was previously composed of 3 species from China and Taiwan, and characterized only by wing pattern. This species group is extensively studied herein with two new species described, i.e. *P. viridiflamma* **sp. n.** (Taiwan) and *P. minwangi* **sp. n.** (S. China), and discovery of female genitalia of three species, presenting new phylogenetic insights in this potentially paraphyletic genus. In addition, one limacodid larva was found to be feeding exclusively on *Picea* (Pinaceae) in Taiwan. Its identity, *Parasa pygmy* Solovyev, 2010 in *P. undulata* group, is confirmed through matching its COI sequence to the adult. This discovery is also biologically significant because the previous known host breadth of *Parasa* was of polyphagy on various angiosperm plant families. This case, therefore, represents the first record of conifer-feeding behavior in this family as well as the first of specialized herbivory in the genus. Meanwhile, the background match between *Picea* leaves and larval coloration is shared with other *Picea*-feeding insects. This phenomenon is worth of further investigation in the aspect of convergent evolution of crypsis associated with a particular plant.

## Introduction

(1) Systematic problems surrounding the genus *Parasa* Moore, 1859 and its relatives.

The southeastern Asian limacodid moths comprise about 90 species of “green limacodids”, that are green at least on some parts of wings and thorax (Holloway 1986; Solovyev 2011). These species were hitherto all embedded in the genera *Parasa* Moore, 1859 (based on a New World species, *Parasa chloris* Herrich-Schäffer, [1854]) and *Latoia* Guérin-Ménéville, 1844 (based on a Madagascan species, *Latoia albifrons* Guérin-Ménéville, 1844) according to interpretation of morphology, including known female and immature characters (Seitz 1913; van Eecke 1925; Inoue et al. 1982, 1992; Cai 1983; Wu and Fang 2009, 2010; Wu 2011). The taxonomic history of these groups has been detailed in several studies, i.e. Holloway (1986), Holloway et al. (1987) and Solovyev (2011) and the current generic name *Parasa* has been adopted and generally accepted for the Southeast Asian green limacodids since Holloway (1986).

Though a subgrouping of *Parasa* into 14 subunits was proposed by Solovyev and Witt (2009) and Solovyev (2010, 2011) based on the diversity of wing color patterns and differences in genitalia, the potential paraphyly of this genus indicated by Holloway (1986) has not yet been elucidated. This is mainly due to the conservative male genitalia, except in the *Parasa bicolor* species group, as well as limited taxon sampling of female and immature characters. Futhermore, the green pigmental appearance is not only present in *Parasa* and *Latoia*, but also in nine more genera according to Solovyev (2011). In addition, the presence of four black dorsal patches at the posterior marginal scoli of the larva seems to be a potential synapomorphy for *Parasa* (Holloway, 1986). The concatenation of morphological characters (Holloway 1986) and molecular data (Solovyev 2011) are expected to illuminate taxonomic problems surrounding the genus *Parasa*.

(2) Discovery of conifer-feeding habits with unique larval morphology and two additional new species in the *Parasa undulata* species group.

Recently, a single limacodid larva was discovered on the conifer tree Taiwan Spruce (*Picea morrisonicola* Hayata, 1908, Pinaceae), at mid-elevation (ca. 2600 m) of the central mountain range of Taiwan. This presents unusual ground maculation and an external appearance similar to the stomatal band of conifer leaves that is described and discussed in detail below.

Since this larva failed to pupate successfully after the prepupal stage in an indoor rearing environment, we sequenced its mitochondrial COI for comparison to that of two other limacodid moths. These two moths, *Parasa pygmy* Solovyev, 2010 and *Parasa martini* Solovyev, 2010, only occur in mid to high elevation montane regions of Taiwan. These data deposited in Genbank (KF595045, KF595046, KF595047) reveal zero divergence between the collected larva and the adult of *Parasa pygmy*, but ca. 6.3% p-distance divergence to *Parasa martini*, thus confirming the identity of the first known conifer-feeding limacodid (although not unique among zygaenoids, e.g. *Psycharium* Herrich-Schäffer, 1856, Somabrachyidae feeds on *Pinus*: Epstein et al. 1999). Accompanying our larval discovery, we also describe two additional montane species, *Parasa viridiflamma* sp. n.and *Parasa minwangi* sp. n. The above four moth species all belong to the *Parasa undulata* species group as defined by Solovyev (2010, 2011). In addition, the female genitalia have been regarded as including important characters to separate the genus *Parasa* and the Madagascan *Latoia*. In the present study, all females and the genitalia of Taiwanese species are described for the first time. These results provide new evidences to reassess the relationships between the species groups of *Parasa* or the so-called “green limacodids” globally.

## Materials and methods

### Specimen acquisition

The studied specimens were examined in or borrowed from the following institutions and private collections:

BMNH The Natural History Museum, London

CCMF Collection of Chien-Ming Fu, Taichung

CVAK Collection of Valentin A. Kalinin, Moscow

ESRI Taiwan Endemic Species Research Institute, Nantou

NMNS National Museum of Natural Science, Taichung

NSMT National Museum of Nature and Science, Tsukuba

SCAU Entomological Department, South China Agricultural University, Guangzhou

TFRI Insect collection of Taiwan Forestry Research Institute, Taipei

### Genitalia preparations for morphological studies

Genitalia were prepared following the general method described e.g. by Holloway et al. (1987) with slight modification. After maceration of the abdomen in 10% KOH and subsequent cleaning, male genitalia were carefully removed from the abdomen and abdominal segments 1–8 were opened along the caudocephalic axis from the right side. Female genitalia were removed entirely from the abdomen, cleaned and mounted with the ventral side uppermost. All the chitinuous genital tubes, including bursae, derived from the genital openings were preserved. Genitalia and abdominal skins of both sexes were stained with pen ink (Pilot), preserved in 70% ethanol then transferred in 99.5% ethanol before mounting in Euparal on slides. Specimens were photographed using a Nikon D300 digital camera.

### Terminology

The terminology of wing patterns and genital structures follows that of Solovyev (2011), that of immature morphology follows Epstein (1996).

### Molecular analysis

Genomic DNA was extracted from fragments of adult legs and part of larval tissues using an ALS Tissue Genomic DNA Extraction Kit (Kaohsiung, Taiwan). A partial COI sequence was amplified by a polymerase chain reaction (PCR) with a set of universal primers (LCO1490 and HC02198) (Folmer et al. 1994). The PCR was initiated at 95°XC for 5 min, followed by 35 cycles at 95°C for 1 min, 40°C for 1 min, and 72°XC for 1 min, with a final extension at 72°XC for 7 min. The PCR products were separated by electrophoresis in 1.5% agarose gels and sequenced. The 636 nucleotide base pairs of high quality COI sequences were aligned using CLUSTALX 2.0.10 (Thompson et al. 1997). Pairwise genetic distances were calculated using MEGA 4.0.2 (Tamura et al. 2007). Three newly sequenced COI data were deposited in the GenBank database (numbers mentioned above) (http://www.ncbi.nlm.nih.gov/genbank/). The voucher specimens were preserved in the Insect collection of Taiwan Forestry Research Institute, Taipei, Taiwan.

## Systematics

*Parasa undulata* species group

- *Parasa viridiflamma* sp. n. (Taiwan)
- *Parasa undulata* (Cai, 1983) (central and southern China)
- *Parasa pygmy* Solovyev, 2010 (Taiwan)
- *Parasa minwangi* sp. n. (S. China)
- *Parasa martini* Solovyev, 2010 (Taiwan)

**Figures 1–9.**
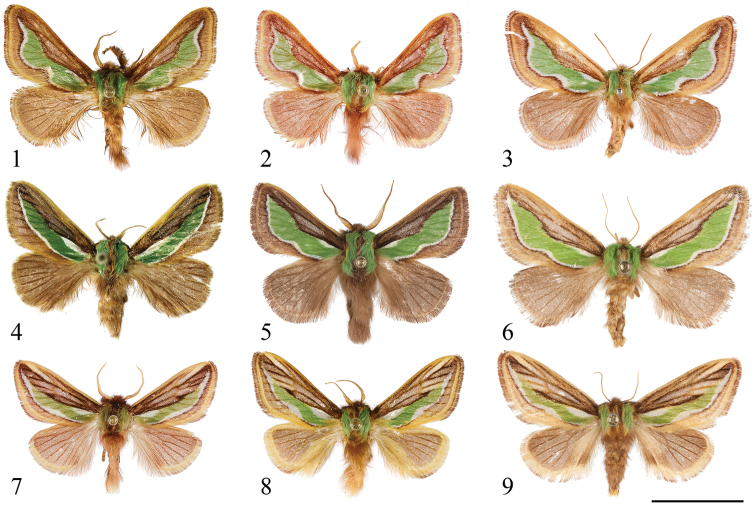
Dorsal views of *Parasa* spp. from China and Taiwan. **1** *Parasa viridiflamma* sp. n., male, paratype, Taiwan **2** *ditto*, male, paratype, Taiwan **3** *ditto*, female, paratype, Taiwan **4** *Parasa undulata* (Cai, 1983), male, Guangxi Province, S. China **5** *Parasa pygmy* Solovyev, 2010, male, Taiwan **6** *ditto*, female, Taiwan **7** *Parasa minwangi* sp. n., male, holotype, Guangdong Province, S. China **8** *Parasa martini* Solovyev, 2010, male, Taiwan **9** *ditto*, female, Taiwan. Bar scale=10 mm. Specimens by courtesy of: NSMT (**1–3, 8**); CVAK (**4**); TFRI (**5, 9**); ESRI (**6**); SCAU (**7**). Photo by Shipher Wu (**1–3, 5–9**); Alexey Solovyev (**4**).

The definition and diagnosis of the *Parasa undulata* species group given in Solovyev (2010, 2011) regarded the median green patch as a likely apomorphic character. Here we re-define this group by following characters:

- Forewing with median green patch surrounded by two white longitudinal stripes, i.e. a short basal stripe and another long one along outer margin of the patch (see Figs 1–9). Notes. The combined pattern of forewings of the resting moths is similar to the needle leaves and stomatal band of several conifer genera, e.g. *Pinus*, *Tsuga* and *Abies* (Pinaceae), thus revealing a potentially adult adaptation to such a resting environment (see Figs 26, 27).
- Corpus bursae with only one transverse signum (see Figs 20–25), rather than two in other species groups of the genus *Parasa*.
- Mature larva, at least that of *Parasa pygmy*, with green ground coloration, white longitudinal stripes and without dorsal abdominal scolus structures. See description part of immature stage of *Parasa pygmy* in detail.

### Key to the species of *Parasa undulata* species group

**Table:** 

1	Forewing green patch wide, extended over approximately half of discal area	2
–	Forewing green patch narrow, covering less than half of discal area; a pale ochreous stripe arising between vein R3 and R4	4
2	Outer margin of green patch distinctly sinuous	3
–	Outer margin of green patch smoothly curved	*Parasa undulata*
3	Outer margin of green patch deeply incised between cubitals and anal vein	*Parasa viridiflamma* sp. n.
–	Outer margin of green patch slightly incised between cubitals and anal vein	*Parasa pygmy*
4	Forewing white stripes wide, terminal and anal areas of forewing with wider ochreous band; anal field of hindwing ochreous	*Parasa martini*
–	Forewing stripes slender, terminal and anal areas of forewing with narrower ochreous band; anal field of hindwing brown, without ochreous coloration	*Parasa minwangi* sp. n.

### Parasa viridiflamma sp. n.

http://zoobank.org/0F20787E-FA13-480F-87C4-9DBC99F06263

http://species-id.net/wiki/Parasa_viridiflamma

**Figures 10–19.**
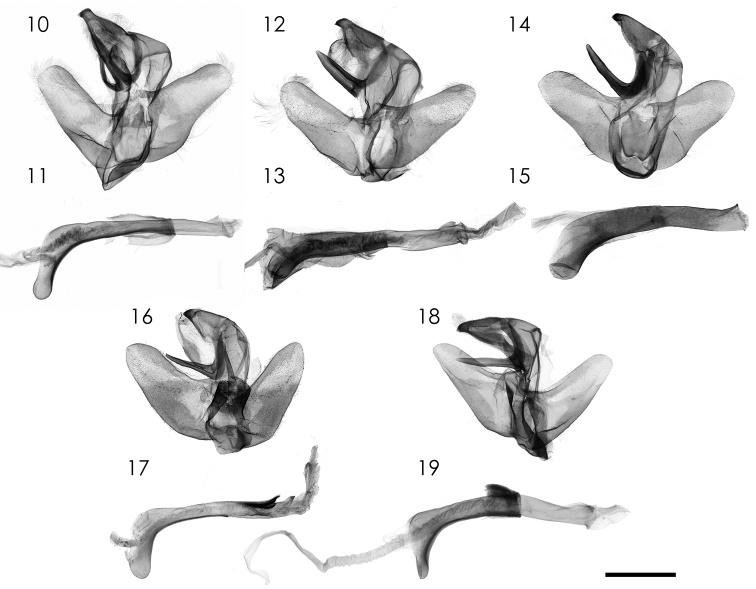
Male genitalia of *Parasa* spp. from China and Taiwan. **10, 11** *Parasa viridiflamma* sp. n., holotype, Taiwan **12, 13** *Parasa pygmy* Solovyev, 2010, Taiwan **14, 15** *Parasa undulata* (Cai, 1983), Guangxi Province, S. China **16, 17** *Parasa minwangi* sp. n., holotype, Guangdong Province, S. China **18, 19** *Parasa martini* Solovyev, 2010, Taiwan **10, 12, 14, 16, 18** Male genital apparatus **11, 13, 15, 17, 19** Aedeagi. Bar scale=1 mm. Specimens by courtesy of: ESRI (**10, 11, 12, 13**); CVAK (**14, 15**); SCAU (**16, 17**); TFRI (**18, 19**). Photo by Shipher Wu (**10–13, 16–19**); Alexey Solovyev (**14, 15**).

**Figures 20–25.**
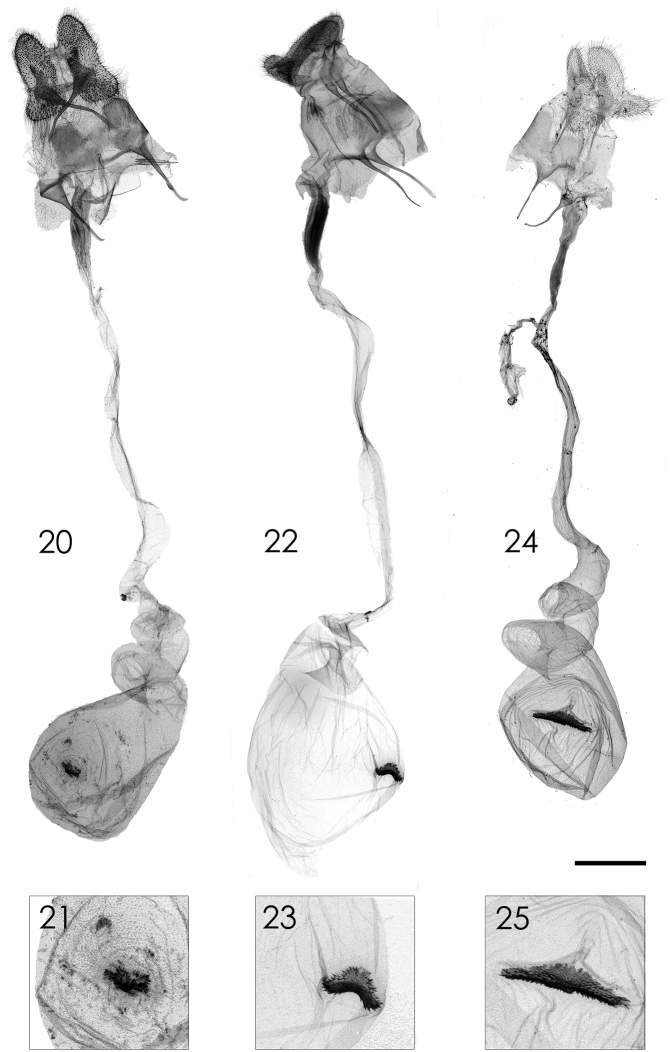
Female genitalia of *Parasa* spp. from Taiwan. **20, 21** *Parasa viridiflamma* sp. n., paratype **22–23** *Parasa pygmy* Solovyev, 2010 **24–25** *Parasa martini* Solovyev, 2010 **21, 23, 25** Magnified images of signa. Bar scale=1 mm. Specimens by courtesy of: NSMT (**20–21**); ESRI (**22–23**); TFRI (**24–25**). Photo by Shipher Wu.

Figs 1–3 , 10 , 11 , 20 , 21

#### Type material.

Holotype: ♂, TAIWAN, Hualien County, Tayuling, 2550 m, 25-VI-2008, leg. H. H. Lin (coll. ESRI); paratypes: 3♂, Taichung County, Tashuehshan Mts., Anmashan, 2230 m, 14-16.VI.1989, leg. M. Owada; 1♂, same collecting data, slide NSMT-SW131; 1♀, Taichung County, Anmashan, 2300 m, 30-VII-1997, leg. T. Mano, slide NSMT-SW132 (coll. NSMT); 1♂, Taichung County, Anmashan, 2600 m, 23-V-1998, leg. C. M. Fu; 1♂, Taitung County, Yenping, 31-VII-1992, leg. Shiau & Yang (coll. NMNS); 1♂, Nantou County, Renluen, 1400 m, 21-VIII-1991, leg. Y. B. Fan, slide TFRI00061358; 1♂, Nantou County, Tatajia, 2610 m, 6-VII-2011, leg. S. Wu & W. C. Chang (coll. TFRI).

#### Diagnosis.

The new species is externally similar to *Parasa undulata* from central and southern China and *Parasa pygmy* from Taiwan but it can be easily distinguished by the forewing green patch strongly incised between cubitals and anal veins. In the male genitalia the basal part of aedeagus (coecum) is long, strongly extending toward ventral side in *Parasa viridiflamma*. Females of all three Taiwanese species of the *Parasa undulata* group are recorded in the present study, they can be distinguished by the shape of the single signum, that of *Parasa viridiflamma* is short, irregular in shape, that of *Parasa pygmy* is saddle-shaped and that of *Parasa martini* is straight and long in transverse axis.

#### Description.

Adult (Figs 1–3).

Measures. Wingspan 23–24 mm in male (n=7); 26 mm in female (n=1).

Head. Antennae bipectinate in male, rami longer at basal part and gradually shortening to absent at 5/6 from base; filiform in female. Eyes black, round. Frons, vertex, labial palpi fringed with long, chestnut hair-like scales, 3rd labial palpal segment short.

Thorax. Thoracic segments green with chestnut dorsal stripe. Forewing ground coloration chestnut with median large green patch delimited externally by white line which is in turn lined by brown border, all these pattern elements strongly incurved between cubitals and anal veins, less so towards termen; marginal scales ochreous. Hind wings chestnut, marginal scales ochreous.

Abdomen. Abdominal segments fringed with long chestnut hair-like scales.

Male genitalia (Figs 10, 11). Uncus robust, wide with hook-like apex. Gnathos. Gnathos large, sclerotized, apically narrowed; juxta plate-like with two lateral sides extending dorsally. Valva short, apex tongue-like. Aedeagus long, tubular, coecum strongly bent ventrally.

Female genitalia (Figs 20, 21). Apophyses elongated, length of anterior and posterior ones equal; ductus bursae long; corpus bursae small, about 3.5 times shorter than ductus bursae, signum small, irregular-shaped.

#### Distribution and bionomics.

This species is endemic to Taiwan. The adults occur in May and mid June to late August in mid-elevation mountain areas (1400–2610 m). The fresh individuals appear earlier in the season. Possibly univoltine. Hostplant unknown.

#### Etymology.

The new species is named through the combination of *viridis* (green) and *flamma* (flame), according to its flame-shaped green median patch on forewing.

### Parasa pygmy

Solovyev, 2010

http://species-id.net/wiki/Parasa_pygmy

Figs 5 , 6 , 12 , 13 , 22 , 23 , 26–33

Parasa pygmy Solovyev, 2010, 89 (11): 1358, figs. 1, 5; 2, 5; Solovyev 2011, 91(1): 100, figs. 1, 5; 2, 5. (Type locality: Taiwan)

#### Material examined.

TAIWAN, 2♂, Chiai Hsien [Nantou County], Luhlin Lodge, [ca. 2600 m], 16-VIII-1990, leg. B. S. Chang (coll. NMNS); 3♂, Miaoli County, Guanwu, 2000 m, 27-IX-2010, S. Wu leg.; 1♂, Nantou County, Black Water Cottage, 2757 m, 7-IX-2012, S. Wu & W. C. Chang leg.; 1♂, Nantou County, Chen-gong Lodge, 2853 m, 10-IX-2012, leg. S. Wu & W. C. Chang; 7♂, Nantou County, Piluchi, 2000 m, 3-IX-1986, leg. Y. J. Chang; 4♂, same locality, 4-IX-1986, Y. J. Chang leg.; 18♂, same locality, 14-IX-1986, Y. J. Chang leg.; 5♂, same locality, 15-X-1987, Y. B. Fan (coll. TFRI); 1♂, Nantou County, Hohuanshan, 3006 m, 14-IX-2009, L. C. Shih leg., slide ESRI A12-20090914-037 (coll. ESRI); 1♂, Nantou County, Yuanfeng, 2700 m, 11-IX-2012, leg. S. Wu, slide TFRI00148804; 1♂, Nantou County, Xiaofengko, 3002 m, 13-VIII-2012, leg. S. Wu & W. C. Chang; 1♂, Ilan County, Jianchin, 1930 m, 8-X-2012, leg. S. Wu; 8♂, Hualien County, Guanyuan, 2400 m, 13-IX-2012, leg. S. Wu (coll. TFRI); 13♂, Hualien County, Kuanyan (=Guanyuan), 2370 m, 13-IX-2012, leg. M. Owada & S. Wu (coll. NSMT); 1 Mature larva, Hualien County, 820 Logging Trail, 2600 m, 26-V-2012, leg. S. Wu & W. C. Chang (coll. TFRI); 1♀, Hualien County, Jinma Tunnel, 2400 m, 23-IX-2009, leg. L. C. Shih, slide ESRI A09-20090923-127 (coll. ESRI); 1♀, Hualien County, Biluishenmu, 2150 m, 22-VIII-1991, leg. H. Y. Wang (coll. NMNS).

#### Diagnosis.

This species represents the insular sister species of *Parasa undulata* from China. It can be easily distinguished from *Parasa undulata* by its broader forewing medial green patch and its longer coecum. The comparison of the female genitalia is given under the diagnosis of the preceding species.

#### Description.

The female and mature larva are described for the first time.

Female (Fig. 6).

Measures. Wingspan 24–25 mm (n=3).

Head. Antennae filiform. Eyes black, round. Frons, vertex, labial palpi fringed with long, chestnut hair-like scales, 3rd labial palpal segment short.

Thorax. Thoracic segments green with chestnut dorsal stripe. Forewing ground coloration chestnut with large median green patch delimited externally by thin white line which is in turn lined by brown border; marginal scales ochreous. Hind wings chestnut, marginal scales ochreous.

Abdomen. Abdominal segments fringed with long chestnut hair-like scales.

Female genitalia (Figs 22–23). Apophyses elongated, length of anterior and posterior ones equal; ductus bursae long; corpus bursae small, about 3.5 times shorter than ductus bursae, signum saddle-shaped in transverse axis.

Immature stages.

Mature instar (Figs 28–33). Body spindle-like, length 20 mm when fully extended. Legs very small, largely reduced. Prolegs fully absent; adhesive, sucker-like regions on abdomen present. Head and body ground coloration green; a pair of prominent conical dorsal scoli arising from the dorsal part of mesothorax and on the 9th abdominal segment, respectively, the remaining parts smooth. 10 fresh red spots, circled by light blue ring, arranged longitudinally along mid-dorsum; two cream yellow subdorsal lines running parallel adjacent to the red spots; dorso-lateral, lateral and ventro-lateral lines wide; regions between subdorsal and dorso-lateral lines pale green; small subdorsal scoli, arising from mesothorax, metathorax and abdominal segment A2 to A8, orange, along on lateral lines and reduced as small scobinate patches; spiracles orange.

**Figures 26–33.**
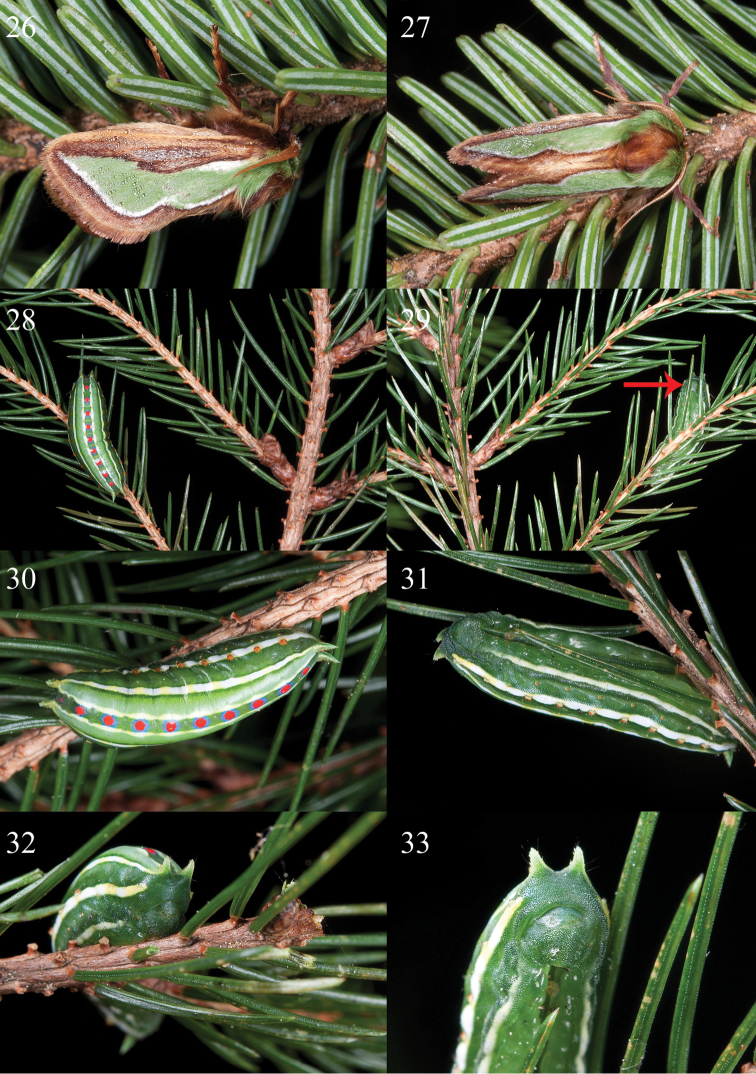
Photos of alive *Parasa pygmy* Solovyev, 2010 in Taiwan. **26, 27.** Adult male on *Abies kawakamii* (Pinaceae) **26** Lateral side **27** Dorsal side **28–33** Mature instar on hostplant, *Picea morrisonicola* (Pinaceae) **28** Resting posture on ventral side of hostplant **29** *ditto*, dorsal view, denoted by red arrow **30** Magnified image **31–33** Larva feeding on leaf. Photo by Shipher Wu.

#### COI sequence.

Two new COI sequences (identical) from adult male and mature larva, respectively, were deposited in the GenBank database (KF595046, KF595047).

#### Distribution and bionomics.

*Parasa pygmy* is endemic to Taiwan. The adults occur from mid August to early October in mid to high elevation mountains of central Taiwan (~2000–3000 m), where they match the distribution range of the presently known hostplant, *Picea morrisonicola* Hayata (Pinaceae). The single mature larva was taken in late May, the leaf flushing period of *Picea morrisonicola*. This observation suggests a univoltine life cycle for *Parasa pygmy* and the overwintering stage is inferred to be the egg. The patterns of the adult and mature larva are similar to the needle leaves of Pinaceae, especially the hostplant species. This potential evolutionary adaptation is detailed in results and discussion.

### Parasa undulata

(Cai, 1983)

http://species-id.net/wiki/Parasa_undulata

Figs 4 , 14 , 15

Latoia undulata Cai, 1983, 4 (26): 449, fig. 10. (Type locality: Dukou, Sichuan [China])
Parasa undulata : Solovyev 2010, 89 (11): 1358; Wu and Fang 2010: 43, pl. 18, fig. 1–36; Solovyev 2011: 100.

#### Material examined.

CHINA, 1♂, Guangxi Province, Dayao Shan Mts. Jingxiu, 100 km SE Liuzhou, 24°07'N, 110°14'E, 1700 m, VII-2008, leg. V. Siniaev (coll. CVAK).

#### Diagnosis.

This species is closely related to *Parasa pygmy*, their comparison is given under the diagnosis of preceding species.

#### Distribution and bionomics.

According to Wu and Fang (2010), this species is widely distributed in China (Henan, Anhui, Hubei, Sichuan, Yunnan, Shaanxi and Gansu). It is recorded in Guangxi Province for the first time. Hostplant unknown.

### Parasa minwangi sp. n.

http://zoobank.org/F5D21EB2-EB61-42D4-953C-48F8478E2EB5

http://species-id.net/wiki/Parasa_minwangi

Figs 7 , 16 , 17

#### Type material.

Holotype: ♂, CHINA, Guangdong Prov., Shaoguan, Nanling, 700-1200 m, 22-25-IV-2005, leg. K. Horie, slide NSMT-SW133 (coll. SCAU); paratypes: 4♂, Guangdong Prov., Shaoguan, Nanling, 600–1400 m, 11-18-V-2005 (coll. NSMT); 4♂, same collecting data (coll. SCAU); 1♂, same collecting locality, 21-28-VI-2008 (coll. NSMT); 2♂, same collecting locality, 1-6-VIII-2006 (coll. NSMT); 1♂, same collecting data (coll. SCAU); 1 male, same collecting locality, 31-VIII-1-IX-2003 (coll. NSMT); 1♂, same collecting data (coll. SCAU); 2♂, 5-11-IX-2005 (coll. NSMT); 2 males, same collecting data (coll. SCAU); 1 male, 26-27-IX-2003, leg. M. Wang et al. (coll. NSMT); 1 male, same collecting data (coll. SCAU), all leg. Wang et al.

#### Diagnosis.

This species is closely related to the allopatric species *Parasa martini* from Taiwan. Externally its forewing white stripes are more slender. In the male genitalia the aedeagus has a more slender coecum and bears a separated, sclerotized dorsal process at the apex, the latter structure being absent in other species of the same group.

#### Description.

Adult (Fig. 7).

Measures. Wingspan 21–22 mm (n=21).

Head. Antennae bipectinate in male, rami longer at basal part and gradually shortening to absent at 5/6 from base; filiform in female. Eyes black, round. Frons, vertex, labial palpi fringed with long, chestnut hair-like scales, 3rd labial palpal segment short.

Thorax. Thoracic segments green with chestnut dorsal stripe. Forewing ground coloration chestnut with ochreous stripe situated between vein R4 and R5 and a large median green patch delimited by slender white lines and subsequent wide brown border; marginal scales ochreous. Hind wings chestnut, marginal scales pale chestnut fringed with ochreous.

Abdomen. Abdominal segments fringed with long chestnut hair-like scales.

Male genitalia (Figs 16, 17). Uncus robust, wide with hook-like apex. Gnathos large, sclerotized, apically narrowed; juxta sclerotized with two lateral sides extending dorsally. Valva short, apex tongue-like. Aedeagus long in straight distal part and down-curved basal part, respectively, and with a distal sclerotized dorsal process.

#### Distribution and bionomics.

This species is recorded only from mid-elevation (600–1400 m) of Nanling mountain areas, S. China. The adults occur in April, May, June, August and September. Possibly bivoltine. Hostplant unknown.

#### Etymology.

This species is dedicated to Dr. Min Wang (SCAU), who represents the main collector of most of the type material of this new species in Nanling mountain areas, S. China.

### Parasa martini

Solovyev, 2010

http://species-id.net/wiki/Parasa_martini

Figs 8 , 9 , 18 , 19 , 24 , 25

Parasa martini Solovyev, 2010, 89(11): 1358, figs. 1, 6; 2, 6; Solovyev 2011, 91(1): 100, figs. 1, 6; 2, 6. (Type locality: Taiwan)

#### Material examined.

**Type material:** Holotype. ♂, “TAIWAN, Taichung County., He-ping, Dayueshan National Forest Recreation Area, N24°15.315 E121°00.374, 28-V-2007, 2223 m, At MV light, leg. G. Martin & D.L.J. Quicke, BMNH (E) 2007-43”,”BMNH (E) # 820958”,”BMNH genital slide 1422” (coll. BMNH), paratypes: 2♂, same collecting data as holotype (coll. BMNH)

**Other material:** TAIWAN, 1♂, Miaoli County, Guanwu, 2000 m, 29-VI-2010, leg. S. Wu & W. C. Chang; 1♀, same collecting data, slide TFRI00143030 (coll. TFRI); 1♂, Taichung County, Anmashan, 2100 m, 19-VIII-1996, leg. C. M. Fu; 4♂, same collecting locality, 13-IX-1996, leg. C. M. Fu; 1♂1♀, same collecting locality, 30-VI-1997, leg. C. M. Fu (CCMF); 1♂, same collecting locality, 2200 m, 29-VII-1997, leg. C. M. Fu; 1♂, Baxianshan, 1000 m, 26-VII-1997, leg. C. M. Fu (coll. CCMF); 3♂1♀,Taichung County, Chingshan, 1400 m, 9-10-IX-1993, leg. W. T. Yang & M. L. Chan (coll. NMNS); 3♂, Taichung County, Wushihken, 950 m, 23-V-2012, leg. L. C. Shih (ESRI); 1♂, Nantou County, Piluchi, 2000 m, 12-VIII-1987, leg. Y. B. Fan, slide TFRI00061365; 1♂, Hualien County, Ci’en, 1950 m, 13-IX-2012, leg. S. Wu (coll. TFRI); 1♂, Hualien County,Cien, 2039 m, 20-VII-2009, leg. L. C. Shih (coll. ESRI); 1♂, Hualien County, Tsuen (=Ci’en), 2000 m, 13-IX-2012, leg. M. Owada & S. Wu (coll. NSMT).

#### Diagnosis.

This species is the allopatric sister-species of *Parasa minwangi* sp. n. from southern China. Their comparison is given under the diagnosis of the preceding species.

#### Description.

The female is described here for the first time.

Female (Fig. 9).

Measures. Wingspan 24–25 mm (n=3).

Head. Antennae filiform. Eyes black, round. Frons, vertex, labial palpi fringed with long, chestnut hair-like scales, 3rd labial palpal segment short.

Thorax. Thoracic segments green with chestnut dorsal stripe.

Forewing ground coloration chestnut with ochreous stripe situated between vein R4 and R5 and one median longitudinal green patch delimited by white lines and subsequent wide brown border; marginal scales ochreous. Hind wings chestnut, anal margin and marginal scales ochreous.

Abdomen. Abdominal segments fringed with long chestnut hair-like scales.

Female genitalia (Figs 24, 25). Apophyses elongated, length of anterior and posterior ones equal; ductus bursae long; corpus bursae small, about 3.5 times shorter than ductus bursae, signum transverse, long with medial part more expanded.

#### COI sequence.

A new COI sequence was deposited in the GenBank database (KF595045).

#### Distribution and bionomics.

This species is endemic to Taiwan. The adults occur from late May to late June, mid and late July then mid August to mid September in mid-elevation mountain areas with primary vegetation (ca. 950–2223 m). Possibly bivoltine. Hostplant unknown.

## Results

The present study reports on the first record of a conifer-feeding limacodid moth on the Taiwan Spruce (*Picea morrisonicola*) in Taiwan, describing the specialised morphology of the last larval instar. The larval identity is confirmed through COI sequence (636 bp) comparison between *Parasa martini* and *Parasa pygmy*. The sequences of adult and larval *Parasa pygmy* are identical but about 6.3% divergent to *Parasa martini*.

The conifer-feeder, *Parasa pygmy* (Taiwan), together with *Parasa undulata* (central and southern China), *Parasa martini* (Taiwan), and the newly described *Parasa viridiflamma* sp. n. and *Parasa minwangi* sp. n., form the *Parasa undulata* species group sensu Solovyev (2011). The female and genital structures of the Taiwanese species are firstly illustrated and described before discussing the phylogenetic affinity with congeneric species.

## Discussion

Previously known *Parasa* larvae are mostly regarded as being polyphagous, often as agricultural pests on broad-leaved trees (Robinson et al. 2001, 2002) and possess the potentially synapomorphic character of four black dorsal scolus patches on their posterior margin (Holloway 1986). The presence of dorsal and dorsal-lateral scoli places them in the first limacodid group as defined by Solovyev and Witt (2009: 40). In the present study, the first record of a conifer-feeding limacodid moth, *Parasa pygmy*, together with description of the particular morphology of the mature instar is reported. This finding does not likely represent an occasional circumstance of a much broader host plant repertoire since external patterns of the adult and mature larva of *Parasa pygmy* are similar to the needle leaves of Pinaceae species, especially that of its hostplant *Picea morrisonicola*, revealing the tight specialization of the larva at least on Pinaceae. Conifer-feeding macrolepidopteran species in temperate regions that have larval patterns matching those of their hostplants were also reported and illustrated in several studies, e.g. the Japanese *Sphinx caliginea* (Butler, 1877) (Sphingidae) (Nakatomi 1987; Miyata 2011), *Alsophiloides acroama* (Inoue, [1944]) (Geometridae) (Nakajima 2011), the northern American *Lithophane lemmeri* (Barnes & Benjamin, 1929), *Xestia badicollis* (Grote, 1873) and *Feralia comstocki* (Grote, 1874) (Noctuidae) (Maier et al. 2011). This phenomenon is worth of further investigation in the aspect of convergent evolution of crypsis associated with a group of particular plants.

Additionally, the larval habits of *Parasa pygmy* are also interesting. The observed larva always moves and feeds on the ventral side of needle leaf and branches, thus, its patterns and behavior can be regarded as a case of countershaded crypsis on Pinaceae to prevent predation by high mountain birds (lizard species occur more rarely in this high altitude biotope of Taiwan). Although it is cryptic, the prominent red spots of the mid-dorsal line of the mature larva, in contrast to its green ground coloration, act as a potentially aposematic signal (a similar pattern occurs in some *Pinus* feeding Bombycoidea, such as *Lapara bombycoides* Walker, 1856, Sphingidae). The combination of visual crypsis and aposematism was reported in previous studies, e.g. Edmunds (1974), Rothschild (1975), Papageorgis (1975), Endler (1978); Ruxton et al. (2004); Tullberg et al. (2005) and Gamberale-Stille et al. (2009), and this kind of function can be modulated according to the distance between prey (signal) and predator (observer).

In addition to the descriptions of new species and the discovery of conifer-feeding larval habits, the female of three representatives of the *Parasa undulata* species group in Taiwan are reported for the first time. Their genitalia are different to those of other *Parasa* species groups by the presence of only one signum rather than two. The number of signa is hitherto regarded as a significant character state to distinguish a broad sense pantropical *Parasa* from Madagascan *Latoia*. The latter has no signum.

Though the known immature and female characters of the *Parasa undulata* species group show remarkable differences compared to the other *Parasa* species groups, the characters of wing venation and male genitalia are typical for the genus. Therefore, we hesitate to treat this lineage as an independent taxonomic unit until the mentioned characters can be comprehensively analyzed in all green limacodid groups.

In addition, the species richness and distribution of the *Parasa undulata* species group is extensively reviewed in this study, comprising wide-distributed *Parasa undulata* and local-ranged *Parasa minwangi* sp. n. in Asian continental region (China) and three endemic species found in a mountainous island (Taiwan). Assuming that no more or a few undescribed species may be discovered in the mainland in future studies, their distribution patterns show a rather higher species diversification in a small biogeographic unit. Though Holloway and Hebert (1979) claimed the lower opportunities for specialization of conifer-feeding lepidopteran larvae compared to angiosperm-feeding ones, the hostplant selection of different conifer genera is still regarded as a potential key to speciation of this lineage which would be interesting to further investigate.

## Supplementary Material

XML Treatment for Parasa viridiflamma

XML Treatment for Parasa pygmy

XML Treatment for Parasa undulata

XML Treatment for Parasa minwangi

XML Treatment for Parasa martini
